# Supplementary material for: The genome sequence of the Hagfish, Myxine glutinosa Linnaeus, 1758 (Myxiniformes: Myxinidae)
Source: Wellcome Open Res. 2025 Oct 3;10:537. [Version 1] doi: 10.12688/wellcomeopenres.24925.1 (PMC12699212; doi:10.12688/wellcomeopenres.24925.1)
Supplement: Peer review history

# Reviewer report: Reviewer response for version 1

**DOI:** 10.21956/wellcomeopenres.27455.r139923

**Published:** 2025-12-11

**Authors:** Ivan Rodrigo Wolf

**Affiliations:** 1 UNC Charlotte Department of Biological Sciences, Charlotte, North Carolina, USA

The manuscript describes the genome assembly of *Myxine glutinosa*, the Atlantic hagfish. *M. glutinosa* belongs to the order Cyclostomini, the sister group to *Gnathostomi* (the jawed vertebrates), and plays important ecological roles, being a scavenger that contributes to nutrient cycling and oxygenation of the seabed.

 The biomolecule extraction and preparation protocols follow the high standards of the Wellcome Sanger Institute's (WSI) Tree of Life program, a program for investigating life using DNA sequencing, and appear suitable for generating a high-quality reference genome for the species.

 I would just like to suggest that the authors clarify one point further: in the "Methods" section, under the "Genome assembly" first paragraph the authors state " *(...) was generated from the filtered reads using FastK*". However, no filtering methodology is mentioned at this point in the manuscript, a minor inconsistency that is easy to correct.

 Overall, the genome assembly and quality metrics are excellent, given that differences in " *k*-mer completeness" and "Percentage of assembly assigned to chromosomes" values ​​compared to the benchmark in Table 4 are expected.

 The manuscript is suitable for indexed given its proposal to generate a reference genome for the species.

Are sufficient details of methods and materials provided to allow replication by others?

Yes

Is the rationale for creating the dataset(s) clearly described?

Yes

Are the datasets clearly presented in a useable and accessible format?

Yes

Are the protocols appropriate and is the work technically sound?

Yes

Reviewer Expertise:

My specialization is in bioinformatics, with an emphasis on systems biology, genomics, transcriptomics, molecular phylogenetics, immunogenomics, pattern recognition, and the search and characterization of genes of biotechnological interest.

I confirm that I have read this submission and believe that I have an appropriate level of expertise to confirm that it is of an acceptable scientific standard.

# Reviewer report: Reviewer response for version 1

**DOI:** 10.21956/wellcomeopenres.27455.r139931

**Published:** 2025-11-25

**Authors:** Oliver Berry

**Affiliations:** 1 CSIRO, Crawley, Australia

**Overarching comments.**

 Thank you – a useful piece of work on a fascinating creature.

1. Although this is a data note, and thus largely about making a resource available, I felt that the authors could have provided a broader context for the value this particular resource represents. For example, indicating what other basal fish groups have genomes available, whether there are genomic features unique to these groups, whether provision of this genome is proposed to address specific knowledge gaps. This need not be detailed, but I believe would: a) improve readability; and b) strengthen the justification for the work.
2. Again, although it’s a data note, some high-level reflection on the nature of the genome in relation to biological and evolutionary context and expectations would be welcomed by this reviewer. Similarly, I believe that some reflection on the assembly metrics versus benchmarks (Table 4) and how to interpret the generally lower values in relation to benchmarks would be valuable. Perhaps the differences are trivial. Perhaps it’s clear to the authors how genome assembly metrics could be improved in the future. Again, these could be high level reflections, but I believe could help others in the field understand how to approach similar problems.

**Key questions for a Data Note:**

- Is the rationale for creating the dataset(s) clearly described?

The rationale is briefly explained in terms of understanding early evolution of jawed fishes. I would have liked to have heard about existing genomic data relevant to this question, and where provision of this genome would contribute.

- Are the protocols appropriate and is the work technically sound?

I am not an expert on genome sequencing and assembly, but the analysis was thorough and provision of results adjacent to benchmark values was helpful.

- Are sufficient details of methods and materials provided to allow replication by others?
- Yes, they are thorough and links to additional information and resources are indicated.
- Are the datasets clearly presented in a useable and accessible format?

Yes, while presenting genome data can be challenging, given the scale, and richness, I appreciated the detailed figure captions supplied with figures.

Are sufficient details of methods and materials provided to allow replication by others?

Yes

Is the rationale for creating the dataset(s) clearly described?

Partly

Are the datasets clearly presented in a useable and accessible format?

Yes

Are the protocols appropriate and is the work technically sound?

Yes

Reviewer Expertise:

Environmental genomics.

I confirm that I have read this submission and believe that I have an appropriate level of expertise to confirm that it is of an acceptable scientific standard.

# Reviewer report: Reviewer response for version 1

**DOI:** 10.21956/wellcomeopenres.27455.r135229

**Published:** 2025-10-27

**Authors:** Ferdinand Marlétaz

**Affiliations:** 1 Centre for Life’s Origin & Evolution, Department of Ecology, Evolution & Environment, University College London, London, England, UK

This report describes the sequencing of the hagfish Myxine glutinosa genome using Pacbio Hifi technology and proximity ligation for scaffolding. This species is one of the available hagfish in Europe and its genome would constitute a valuable ressource for research community. The genome assembly procedure is appropriately described and the datasets are acessible (though no annotation was performed). 

 I however, have some reservations regarding this specific genome. 

 - It is the somatic and not the germline genome, while another genome for the same species with the germline has been reported (GCF_040869285.1)

 - the coverage used (30x) appears relatively low to assemble with high confidence an heterozygous (1%) vertebrate genome. This reflects in the k-mer analysis, where the coverage seems insufficient to discriminate  between erroneous and haplotype-related lower-frequency k-mers

Are sufficient details of methods and materials provided to allow replication by others?

Yes

Is the rationale for creating the dataset(s) clearly described?

Yes

Are the datasets clearly presented in a useable and accessible format?

Yes

Are the protocols appropriate and is the work technically sound?

Partly

Reviewer Expertise:

evolution of animal genomes, evodevo, single cell

I confirm that I have read this submission and believe that I have an appropriate level of expertise to confirm that it is of an acceptable scientific standard, however I have significant reservations, as outlined above.
